# Do gestures retain mental associations with their iconic origins, even after they become emblematic? An analysis of the middle-finger gesture among American English speakers

**DOI:** 10.1371/journal.pone.0215633

**Published:** 2019-04-30

**Authors:** Benjamin K. Bergen

**Affiliations:** Department of Cognitive Science, University of California, San Diego, San Diego, California, United States of America; Aix-Marseille Université, FRANCE

## Abstract

What concepts and words do communicative gestures activate in the minds of people who view them? It’s widely believed that many gestures grow from *iconic* origins—they look like what they mean—but also that at some point they may become *emblematic—*conventionalized as culturally agreed-upon symbols. How long do links between physical movements of the body and the things in the world they denote persist in the minds of gesture-users? A pair of experiments asks this question for the Middle-Finger, a cross-culturally recognized obscene gesture. The prevailing view is that the gesture originates in a phallic symbol. Yet it is now predominantly used as an emblematic gesture displaying contempt (among other things). It is currently unknown whether the iconic origins of gestures persist through the emblematic stage in the minds of gesture users. Two experiments tested the hypothesis that viewing the Middle-Finger primes thoughts about penises or the word *penis*. The results showed that the Middle-Finger induced no priming of *penis* compared with control, unlike another obscene penis-representing gesture (Finger-Bang), which did. This suggests that the Middle-Finger no longer activates thoughts of penises in the minds of contemporary American English speakers. Emblematic gestures with iconic origins may undergo historical change not just in the functions they serve but also in the effects they have on the minds of people who use them.

## Introduction

Across cultures and languages, humans use communicative gestures to accompany and replace speech [1]. These gestures vary in terms of their *iconicity* [2]. *Iconic* gestures directly reflect aspects of what they denote through their articulated, visible form, as in pantomime. But many gestures with iconic origins transition over time into *emblematic* gestures [3]. Emblematic gestures, like Thumbs-Up or A-OK, have culturally agreed-upon forms and functions; they can replace words and often have conventional labels. But it is currently unknown, as a gesture’s function changes, whether it also loses its iconic links in the minds of the humans who use it. Emblematic gestures might be bereft of iconic associations for language users—acting as arbitrary conventionalized symbols. Alternatively, iconic associations might survive the transition to emblematicity—in principle, a gesture might both have a culturally agreed-upon form and function, and yet still retain iconic associations to its original basis in the minds of users. The two experiments below ask, for one emblematic gesture, whether its original iconic basis is lost once it transitions to an emblem.

### Iconic and emblematic gestures

Iconic gestures are defined by homology between their form and their denotational meaning or function [2]. These include gestures representing physical objects, motion, or positioning in space, where features of the gesture’s articulation (hand shape, position, movement, etc.) resemble features of the denotation. For instance, using an open palm to represent the oscillating trajectory of a falling leaf would be iconic inasmuch as the shape, orientation, or motion of the hand are homologous to those of the leaf is depicts. Iconic gestures have been the object of substantial interest in gesture research in part because they contrast with the majority of the spoken language that they accompany, which is predominantly non-iconic. Iconic gestures appear to play a special role in development [4]. They may also be useful for speakers in retrieving intended words during speech [5]. Comprehenders integrate them with accompanying language in the construction of mental representations of described objects, like the shape, size, orientation, or trajectory [6–9]. And they also use it to disambiguate speech [10]. For instance, a gesture accompanying the expression *double doors* may successfully disambiguate whether the speaker intends side-by-side French doors or stacked Dutch doors.

Contrasting with iconic gestures, at least in their broad strokes [11], are *emblematic* ones. Emblematic gestures usually have conventional labels, and can appear without speech or can even replace speech. Familiar examples are the Thumbs-Up gesture (closed fist with thumb extended upwards), the Peace gesture (index and middle finger spread with palm oriented away from the body), or the Middle-Finger gesture. Some emblematic gestures vary substantially across cultures. For instance, the A-OK gesture, signifies “OK” in English-speaking contexts but is an obscene gesture in Brazil [2]. Others are more consistent cross-culturally, like gestures pointing to the eye, which reliably encode either an exhortation to or promise of alertness [12].

Emblematic gestures have been argued to often derive from iconic origins. For instance Kendon [13] argues that iconic gestures can be conventionalized as part of a culturally agreed upon code. And indeed, as McNeill argues [3], although the iconicity may no longer play an essential role in determining the gesture’s function (it has become a conventionalized component of a cultural code), nevertheless the iconic element may remain. For instance, the Finger-Cross retains spatial iconicity to the Christian cross, despite its emblematicity.

Yet these characterizations of gestural iconicity are determined analytically, through ad-hoc descriptions of analyst-perceived similarity. It is currently unknown what their cognitive status is. Are the iconic origins of iconic and emblematic gestures retained as active associations in the minds of gesture users? It is possible in principle that emblematic gestures, even those with preserved iconic forms, nevertheless no longer activate their iconic source in the minds of comprehenders when viewed. Indeed, to the extent that they are treated as primarily symbolic acts, emblematic gestures might directly activate their denotation or function, and their original iconicity may be irrelevant to processing. Alternatively, it is possible that gestures preserve iconic associations even when they become emblematic. This possibility is consistent with a body of work showing that iconicity plays a role in word processing, even when words are fully conventionalized symbols in a lexicon [14]. We address this question using a particularly noteworthy emblematic gesture, the Middle-Finger.

### The middle-finger

The earliest records of the extended third digit deployed as an obscene gesture come from ancient Greece [15]. The Middle-Finger gesture made an appearance in the bawdy Greek playwright Aristophanes’s 419 bc play *The Clouds*, in which Strepsiades extends the finger towards Socrates and then proceeds to waggle his penis at him [16]. In Laertius’s *Lives of Eminent Philosophers* (from 330 bc), the philosopher and critic Diogenes expresses disdain for Demosthenes, a prominent Greek statesman and orator, by presenting his middle finger and calling him a demagogue [17]. The Middle-Finger subsequently appeared in Rome, where it was known as the *digitus impudicus*, or “indecent finger.” The emperor Caligula reportedly denigrated his subjects by making them kiss his middle finger rather than his hand [12]. Cassius, one of the subjects so-denigrated, then went on to assassinate the emperor (though the causal role of the Middle-Finger in that series of events remains unclear). In another instance, Augustus Caesar allegedly punished an actor who presented a Middle-Finger to a heckling audience member by banishing him from Rome [18].

In the intervening millennia, the Middle-Finger has spread throughout much of the modern world, and due to the extensive influence of American visual media is by any measure recognized as extensively and as cross-culturally as any obscene gesture. It is also intensely inflammatory; people around the world have been arrested [19], fined [20], and even murdered [21] for using it.

The most commonly cited origin story for the Middle-Finger casts it as a phallic symbol [22]. Strepsiades’ juxtaposition of Middle-Finger and penis is consistent with this interpretation, and present-day gesture interpreters, like anthropologist Desmond Morris, see the detailed morphology of the gesture as representing a phallus, “The middle finger is the penis and the curled fingers on either side are the testicles” [12]. Like the proposed iconic origin stories for many other emblematic gestures [1–3, 23–24], the claim here is that the hand encodes a more or less homologous representations of the penis.

But like other emblematic gestures, even if the Middle-Finger’s origin is iconic, there’s no evidence that it remains so in the minds of modern language users. The proposed resemblances between a finger and a penis aren’t particularly hard to see. But it’s easy to read in iconicity when we know what we’re looking for. Geometrically speaking, many things in the world—like the Middle-Finger and like a penis—are longer in one dimension than in the other two. Yet we wouldn’t want to fall into the trap of labeling everything so proportioned as phallic. So it’s still to be determined—in the mind of a contemporary speaker of English—when a finger is a phallus and when a finger is just a finger. How do we know whether raising a middle finger activates thoughts about penises? This is an instantiation of the more general question: how do we know whether any emblematic gesture still activates iconic mental processes?

## Experiment 1

A first experiment investigated whether exposure to an image of a hand with extended middle finger would increase a viewer’s activation of the word *penis* and related concepts. It used a word-stem completion method [25–26]. In word-stem completion, participants are presented with the beginnings, or stems, of incomplete words, and are asked to fill in the remaining characters. The words they provide are affected by recent experience—they can be primed. Word-stem completion has historically been deployed predominantly as a measure of implicit memory in both neurotypical [27] and impaired populations [28].

Word-stem completion is a useful tool for our purpose because the priming effects it measures dissociate from tests of explicit retention like overt recognition or recall of previously presented items [28–29]. This means that this paradigm can detect unconscious associations. Moreover, word-stem completion is sensitive to prior presentation of both words and images [27, 30]. And there are known to be both conceptual and lexical components to word-stem completion priming [31]. This means that we can use word-stem completion following presentation of gestures to measure unconscious associations that participants have with those gestures. If seeing a gesture activates either specific words or specific concepts, then participants should be more likely to complete stems using associated words.

### Method

#### Participants

All research was conducted with approval of the University of California San Diego Institutional Review Board. Two hundred three participants with IP addresses in the United States and who self-reported as native English speakers with normal or corrected-to-normal vision and hearing enrolled in the online study via Mechanical Turk [32], after indicating informed consent through a button press. Two hundred participants was selected as the target number since Roediger et al. [26] found a large effect with a similar number of participants, but Mechanical Turk oversampled to 203. They received $0.10 for participating in the study, which took an average of two minutes. Five were removed from analysis because they failed to correctly answer a question in the memory task described below. The remaining 198 participants had a mean age of 36.6 (s.d. 12.9); 99 were female.

#### Procedure

After giving informed consent, participants received the following instructions:

In this experiment, you have two tasks.

1. **Memorize pictures**
  They will appear briefly throughout the session. There will be a memory test at the end.
2. **Complete words**.
  You will see clues like this:
  k i t t _ _
  Your job is to type in a completion to the word. In this case, you'd type in the answer: *kitten*. Make sure to answer as quickly and accurately as you can. You only have 15 seconds for each clue.

**Fig 1:**
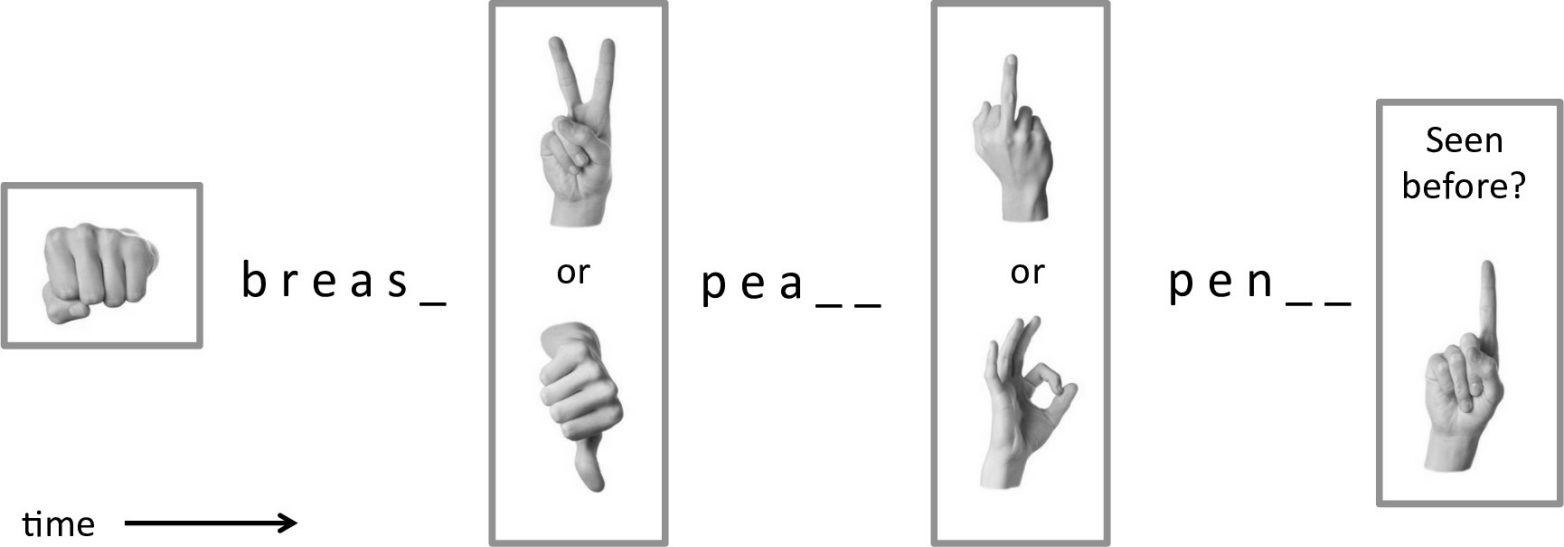
Visual summary of procedure. Images of manual gestures were followed by word-stem completion trials, in which words related to preceding gestures or did not.

The experiment then began. It included only three trials, summarized visually in Fig 1. The first was a practice trials meant to accustom participants to the task and to alert them that the correct answers could be body parts. They saw an image of a fist for three seconds followed by the prompt: b r e a s __ . All but one participant completed this prompt as *breast*, which is the only possible completion in English.

The second trial was a manipulation check—meant to determine whether exposure to a gesture could measurably shift word completion responses in the direction of a word related to the meaning of the gesture. Participants randomly viewed one of two images for three seconds, depicting a Thumbs-Down or a Peace gesture. The subsequent word completion task was: p e a _ _ . Increased *peace* completions instead of *pears* or *pearl*, for instance, after the Peace gesture would suggest that the method was viable.

The final trial was the critical one. Participants randomly saw either an A-OK or a Middle-Finger gesture for three seconds, followed by the prompt: p e n _ _. Increased *penis* responses, as opposed for instance to *penne*, *penal*, or *penny* would be compatible with the hypothesis that the Middle-Finger activates mental representations of the word *penis* or related conceptual knowledge.

After the conclusion of the third trial, participants were presented with a recognition task, in which they were shown one previously unseen image of a hand gesture (a Number-1 gesture) and were asked if they had seen it previously in the experiment. Participants were excluded as described above for responding incorrectly to this trial.

Participants were subsequently asked to report their sex, age, and what they thought the research was about. No other stimuli were presented and no other measures were taken.

#### Materials

Images of hand gestures came from the same archive in a commercial photograph repository and were scaled to approximately 250 x 250 pixels. When it came to the image of the Middle-Finger, there were two common variants to select between—one where all fingers are curled into a fist except for the third digit, and one where the second and fourth digits are straight at the metacarpophalangeal joints, but bent at the proximal interphalangeal joint. To select between them, an Internet image search for “middle finger” was conducted, revealing that—if online images are representative of real-world proportions—the large majority of Middle-Finger gestures are of the former, lone-finger type. There might plausibly be differences in the detailed interpretation of these two variants—perhaps, as Morris suggests, the curled index and ring fingers represent testicles in the minds of gesture users. And yet, if people today interpret these gestures as iconic, the Middle-Finger ought to represent the shaft of a penis in either case, so the more frequent variant was selected.

### Results

#### Manipulation check

**Fig 2:**
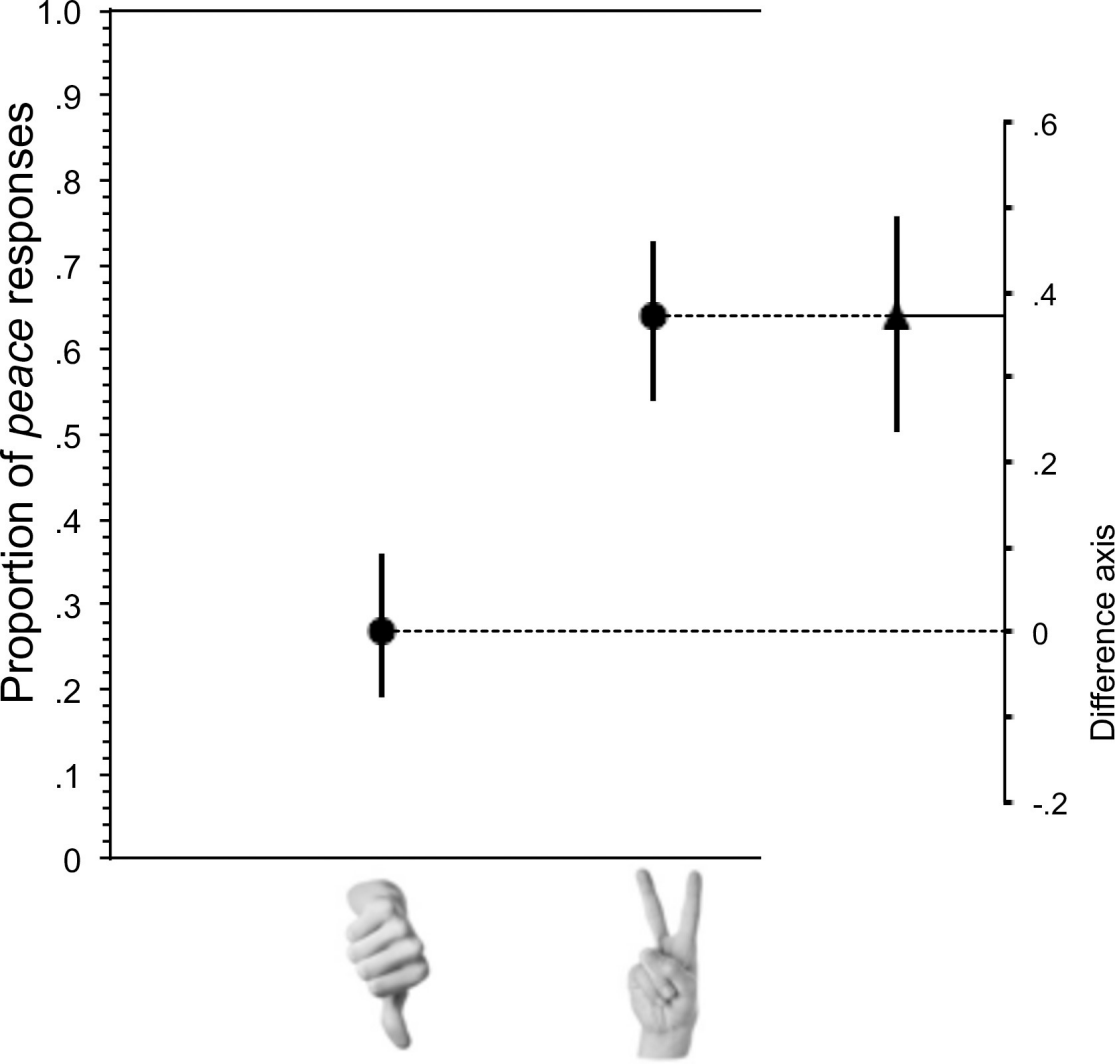
Effect of preceding gesture on completion of word completion prompt: p e a _ _. The proportion of *peace* completions of the p e a _ _ stem was significantly higher following the Peace gesture than the Thumbs-Down gesture. Whiskers indicate 95% confidence intervals. The difference between the group values, with its 95% confidence interval, is shown on a floating difference axis at the right.

Responses to the p e a _ _ prompt were grouped into *peace* or other. Overall, there were 89 *peace* responses and 109 other. This ratio was significantly affected by the gesture image that immediately preceded it, as seen in Fig 2. Specifically, when participants had just seen a Peace gesture, they produced far more *peace* responses (N = 62) than Other (N = 35), while the pattern was reversed following the Thumbs-Down gesture, with *peace* responses (N = 27) were outnumbered by Other responses (N = 74), Fisher’s Exact Test p<0.001. That is, an immediately preceding gesture increases related word completion responses.

Some participants guessed the intent of the experiment. We coded responses to the post-experimental question (“What do you think this research is about, in a few words or a sentence?”) for whether they mentioned any relationship between the gestures and the word completion task. The same analysis above was conducted excluding the 38 participants who mentioned such a relationship. The direction and significance of the effect remained unchanged: Fisher’s Exact Test p<0.001.

#### Critical trial

**Fig 3:**
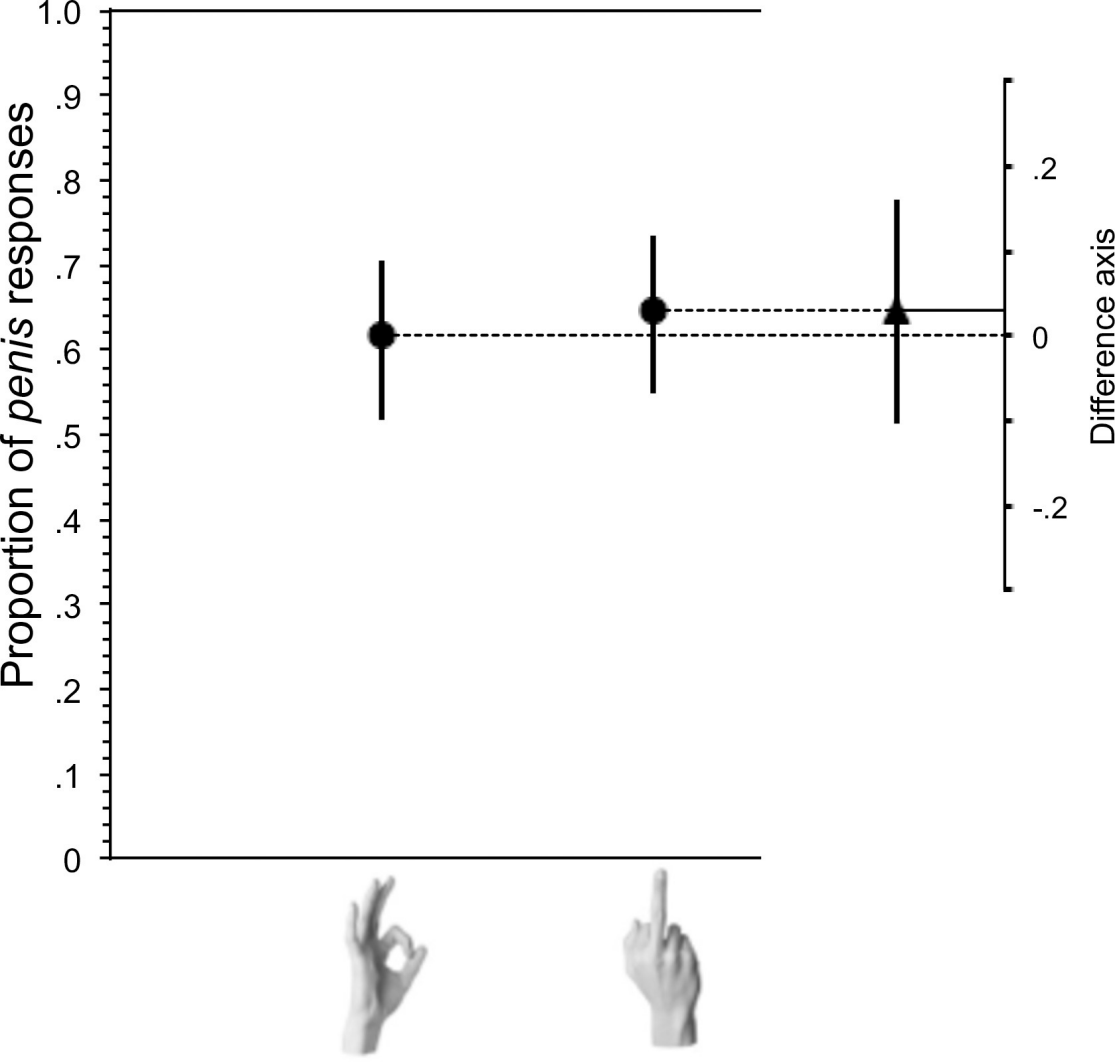
Effect of preceding gesture on completion of word completion prompt: p e n _ _. The proportion of *penis* completions of the p e n _ _ stem was not significantly higher following the Middle-Finger gesture than the A-OK gesture. Whiskers indicate 95% confidence intervals. The difference between the group values, with its 95% confidence interval, is shown on a floating difference axis at the right.

Responses to the p e n _ _ prompt were grouped into *penis* or other. Overall, there were 125 *penis* responses and 73 other. This ratio was not significantly affected by the gesture image that immediately preceded it. When participants had just seen a Middle-Finger gesture, they produced more *penis* (N = 64) than other responses (N = 35), which as seen in Fig 3 was not significantly different from *penis* (N = 61) and other (N = 38) responses following the OK gesture (Fisher’s Exact Test n.s.). This effect was unchanged after excluding the 38 participants who guessed the intent of the experiment, as above: Fisher’s Exact Test n.s. There was no significant difference in the distribution of male and female respondents in the two critical gesture conditions (49 of participants in each condition were male). Age also did not differ significantly between conditions of the critical trial (means = 38.4, 35.8; p>0.1).

### Discussion

The difference between these items is striking. While the Peace gesture significantly primed the word *peace* (demonstrating the viability of this method), no such effect was observed of the Middle-Finger on the word *penis*. One interpretation consistent with this result is that the emblematic Middle-Finger simply does not activate thoughts about penises or the word *penis*.

But there are other possible explanations. Perhaps *penis* responses didn’t increase after the Middle-Finger because of ceiling effects. Because the target word *penis* is taboo, there might be a fixed proportion of the population who will not provide that response, even if they’re thinking about the word. Alternatively, perhaps no effect was observed for the Middle-Finger because the word-stem completion task only detects associations between emblematic gestures and their labels—the Peace gesture is referred to with the label “peace”. The Middle-Finger gesture is obviously not labeled “penis”, so this could explain the difference. A second experiment was designed to adjudicate among these explanations.

## Experiment 2

**Fig 4:**
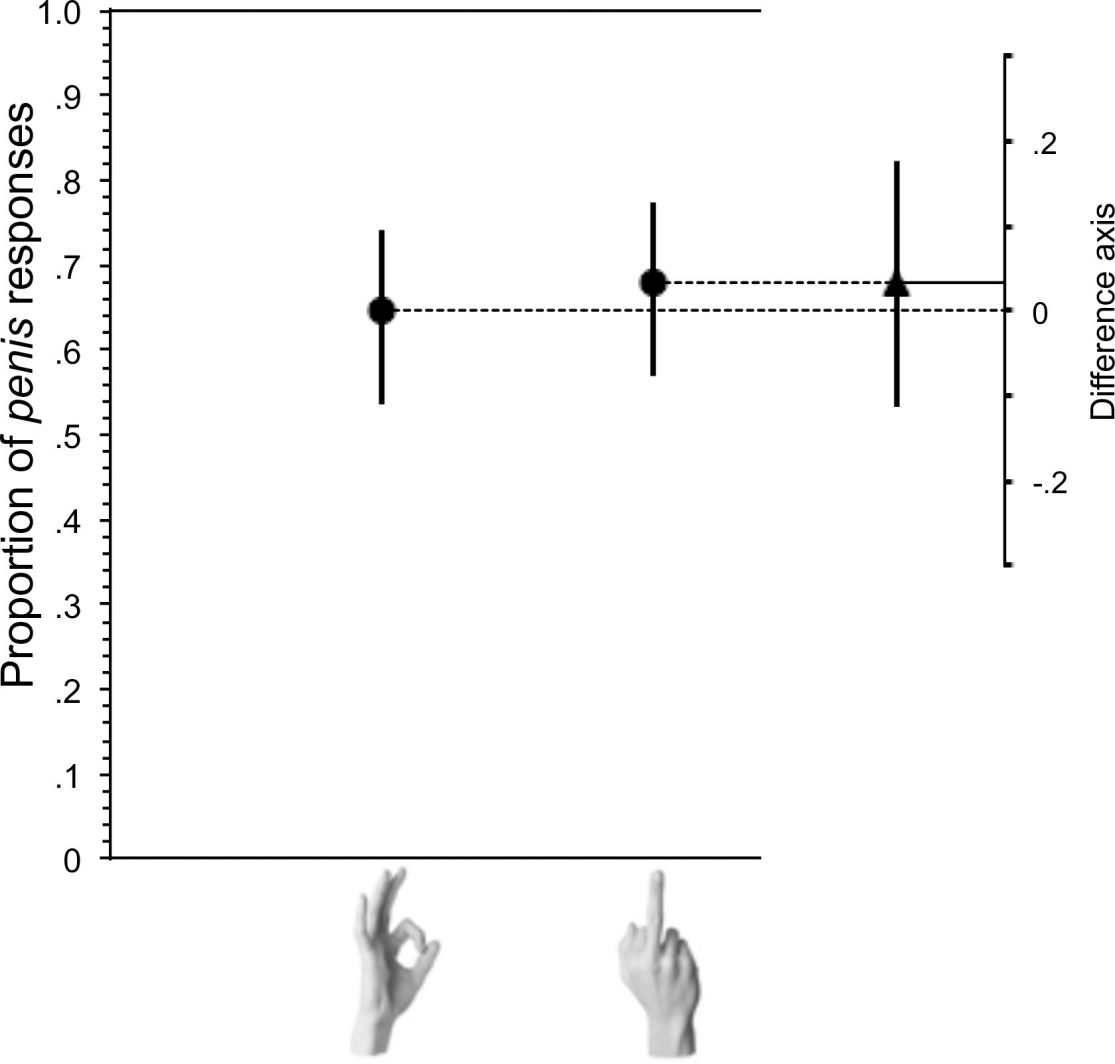
Effect of preceding gesture on completion of word completion prompt: p e n _ _. The proportion of *penis* completions of the p e n _ _ stem was again not significantly higher following the Middle-Finger gesture than the A-OK gesture. Whiskers indicate 95% confidence intervals. The difference between the group values, with its 95% confidence interval, is shown on a floating difference axis at the right.

In order to determine what causes the non-effect of the Middle-Finger gesture on completions of p e n _ _, a third gesture condition was added to the critical trial, using a gesture in which the hand is uniformly agreed to be iconically representing a penis, but for which there is no conventional, emblematic label relating to “penis”. To produce what will be referred to as the Finger-Bang gesture (seen at the bottom right of Fig 4), the index finger of one hand moves inside a loop created by the index and thumb of the other hand.

If neither the Middle-Finger or the Finger-Bang gestures increase *penis* responses to the p e n _ _ prompt, then the method is demonstrably unable to detect activation of penis-related thoughts for gestures of this type (because of ceiling effects or iconicity, or other possibilities). This would mean that we don’t know whether seeing the Middle-Finger increases penis-related thoughts or not. However, if the Finger-Bang gesture does increase *penis* responses but the Middle-Finger still does not, then this would suggest that the method does have the sensitivity to detect penis-related thoughts, and that the Middle-Finger simply doesn’t lead people to think about penises or the word *penis* any more than the A-OK gesture does. And since the Finger-Bang gesture doesn’t have an agreed-upon label—for this reason, it’s not clear it’s even an emblematic gesture at all—this possible difference could be due to iconicity but not label priming.

### Method

This experiment was identical to Experiment 1 with two changes. First, people were randomly assigned to see one of three gestures before p e n _ _ instead of two: A-OK or the Middle-Finger, as before, or Finger-Bang. And second, the target number of participants was increased to 240 due to the addition of one condition. (Mechanical Turk oversampled to 249). Of these, 235 correctly answered the image recognition question and were included in the analysis (95 female; age mean = 31.8, s.d. = 8.6). In all other ways, the experiments were identical.

### Results

#### Manipulation check

Responses to the p e a _ _ prompt were again grouped into *peace* or other. Overall, there were 100 *peace* responses and 135 other. This ratio was significantly affected by the gesture image that immediately preceded it, in the same direction as in Experiment 1. When participants had just seen a Peace gesture, they produced more *peace* responses (N = 72) than Other (N = 45), while the pattern was reversed following the Down gesture, with *peace* responses (N = 28) outnumbered by Other responses (N = 90), Fisher’s Exact Test p<0.0001. Excluding from analysis participants who guessed the intent of the experiment again produced the same result, p<0.001.

#### Critical trial

**Fig 5:**
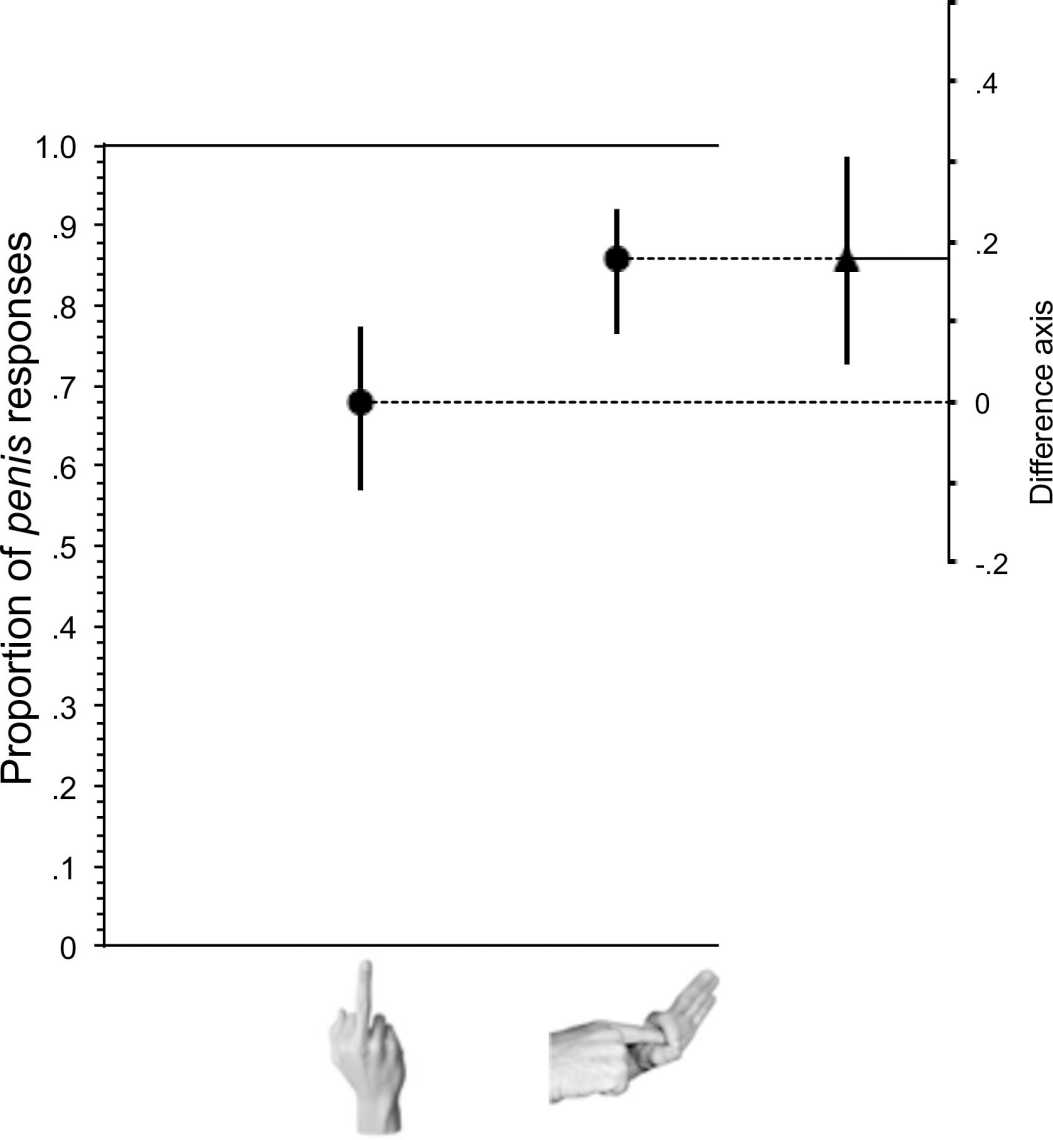
Effect of preceding gesture on completion of word completion prompt: p e n _ _. The proportion of *penis* completions of the p e n _ _ stem was significantly higher following the Finger-Bang gesture than the Middle-Finger gesture. Whiskers indicate 95% confidence intervals. The difference between the group values, with its 95% confidence interval, is shown on a floating difference axis at the right.

Responses to the p e n _ _ prompt were again grouped into *penis* or other. Overall, there were 171 *penis* responses and 64 other. Replicating the result from experiment 1, when participants had just seen an A-OK gesture, they produced more *penis* (N = 51) than other responses (N = 28), which was not significantly different from *penis* (N = 53) and other (N = 25) responses following the Middle-Finger gesture. However, the ratio of penis (N = 67) and other responses (N = 11) following the Finger-Bang gesture differed significantly from responses in the OK gesture condition (p = 0.002) and the Middle-Finger gesture condition (p = 0.006) (Fig 4 and Fig 5). Like in Experiment 1, removing participants who guessed that the experiment was testing for effects of gesture on word completion did not change the outcomes. After excluding the 59 participants who guessed the intent of the experiment, as above, the comparison of OK versus Finger-Bang conditions (p = 0.03) and Finger-Bang versus Middle-Finger (p = 0.005) reached significance, but the comparison of OK and Middle-Finger did not (p = 0.45). There was no significant difference in the ratio of male and female participants assigned to each of the three critical conditions (**𝛘**^2^ = 0.52; *p* = 0.77) or in mean age per condition (means = means = 30.7, 30.5, and 31.1; p<0.1).

In summary, as in Experiment 1, there was no reliable increase in *penis* responses after the Middle-Finger. However, exposure to the Finger-Bang gesture did significantly increase *penis* responses.

## General discussion

In two experiments, images of gestures primed associated word-completion responses—the Peace gesture primed *peace* and the Finger-Bang gesture primed the word *penis*. But the Middle-Finger did not prime *penis*. This suggests that even if the Middle-Finger originated as an iconic representation of a penis in its ancient history, there is no residual evidence of this in the minds of modern gesture users.

A potential concern about the design of the experiment is that the results may have been due to participants consciously reflecting on the nature of the task, in particular the relation between gestures and subsequent words. They then might have strategically vocalized or subvocalized words associated with each gesture image as it appeared, or to seek out words to complete the stems that they were consciously aware related semantically to the preceding image.

But there’s reason to think this isn’t true, or at least that it isn’t necessary to explain the results. As shown in the results above, removing from analysis participants who guessed the structure of the experiment did not change the direction of the reported effects or whether or not they passed traditional thresholds for significance.

### Contemporary cognition does not necessarily recapitulate history

If the Middle-Finger originated as a phallic symbol, there was no trace of it in two experiments. There is some precedent for this finding in the progressive loss or bleaching of historically antecedent meaning for words [33]. As words gain new meanings, their older senses are often lost. Like words, some emblematic gestures appear to migrate beyond their possibly iconic origins. Perhaps as gestures become conventionalized, and as their function shifts, they become less iconic in the minds of users, as proposed by McNeill [3] and hinted at by others [34–37].

It’s worth noting that even if the origin story the Middle-Finger is right, it only goes so far as to explain why people might use an extended finger to represent a penis. But this gesture doesn’t mean “penis.” It serves instead as predominantly a forceful indication of contempt. How this transition took place is a good question in its own right. Anthropologists have argued that it’s just one of many examples where “the act of male erection or copulation becomes symbolic of male dominance and can be used as a dominance gesture in totally non-sexual situations” [12]. If that’s true, it’s hard to recognize in the modern world.

Instead, it’s worth observing that the various obscene gestures around the world find their ostensible origins in things that are themselves taboo: genitalia, sex acts, and so on, just as the obscene words of the world do [38–39]. So the best explanation may be that the same selection pressures that make words about taboo topics most suitable to become profane also take handshapes and body movements about the same topics and groom the best candidates into obscene gestures.

And this may tie in to the gesture’s loss of iconicity. Once a gesture gains a new non-representative function, it may be positioned to lose its iconicity. If the Middle-Finger were used to refer to a penis, and not to demonstrate contempt, then it might be more likely to be interpreted as iconic. Indeed, the Finger-Bang is precisely an example of this in action. Said another way, what differentiates the Middle-Finger from the Finger-Bang—what makes only the latter prime *penis*, may be the fact that the former has moved on in terms of its function from referring to a penis to one of expressing contempt.

In sum, seeing the Middle-Finger doesn’t appear to lead people to think about the word *penis* or about penises in general, and this tells us something about the trajectory of gestures through time. Gestures like the Middle-Finger that derive from imagistic representations take on other functions. The fingers, the fist, and the palm may be selected to represent things that they look like. But their ultimate use is functionally removed from where they originated. This may begin to explain why they harbor no continuing trace of mental iconicity. And the same trend from iconicity to apparently arbitrary conventionality may also be on exhibit in other emblematic gestures, obscene or not [3], iconic signs of signed languages [40], and in the tamed onomatopoeia of spoken languages [41].

## Supporting information

S1 File Data from critical trials in Experiments 1 and 2. De-identified responses for critical trials in each experiment.
(XLSX)
